# Does a working day keep the doctor away? A critical review of the impact of unemployment and job insecurity on health and social care utilisation

**DOI:** 10.1007/s10198-022-01468-4

**Published:** 2022-05-06

**Authors:** Keyi Li, Paula Lorgelly, Sarah Jasim, Tiyi Morris, Manuel Gomes

**Affiliations:** 1 Department of Applied Health Research, Institute of Epidemiology and Health Care, University College London, London, UK; 2 Care Policy and Evaluation Centre, London School of Economics and Political Science, London, UK

**Keywords:** Unemployment, Job insecurity, Health service use, Health care utilisation, I14, I18

## Abstract

While the negative impact of unemployment on health is relatively well established, the extent to which that impact reflects on changes in health and social care utilisation is not well understood. This paper critically reviews the direction, magnitude and drivers of the impact of unemployment and job insecurity on health and social care utilisation across different care settings. We identified 28 relevant studies, which included 79 estimates of association between unemployment/job insecurity and healthcare utilisation. Positive associations dominated mental health services (*N* = 8 out of 11), but not necessarily primary care (*N* = 25 out of 43) or hospital care (*N* = 5 out of 22). We conducted a meta-analysis to summarise the magnitude of the impact and found that unemployed individuals were about 30% more likely to use health services compared to those employed, although this was largely driven by mental health service use. Key driving factors included financial pressure, health insurance, social network, disposable time and depression/anxiety. This review suggests that unemployment is likely to be associated with increased mental health service use, but there is considerable uncertainty around primary and hospital care utilisation. Future work to examine the impact across other settings, including community and social care, and further explore non-health determinants of utilisation is needed. The protocol was registered in PROSPERO (CRD42020177668).

**Supplementary Information:**

The online version contains supplementary material available at 10.1007/s10198-022-01468-4.

## Introduction

The negative impact of unemployment on health and well-being is relatively well established. A previous review [1] has reported a clear detrimental effect of unemployment on an individual’s physical and mental health, after controlling for a wide range of confounding factors and selection issues. The 2010 Marmot Review [2], which outlined a framework for health policymaking in England, concluded that unemployment contributes significantly to poor health and is a major driver of health inequalities. A recent follow-up report [3] highlighted that the impact of job insecurity, such as low-paid, self-employed and short-term employment, on health can be as damaging as that of long-term unemployment.

While unemployment and job insecurity seem to clearly affect health, the extent to which that reflects on changes in health care utilisation is less well understood. First, a large number of studies have reported the direction and magnitude of the impact, but the results are mixed even within the same type of service use and within the same country. For instance, two studies [4, 5] reported contradicting results on the impact of unemployment on hospital admissions in Germany. This suggests that whether unemployment has a true impact on health service use needs to be clarified, to inform policymakers about the health care needs of unemployed individuals.

Second, most studies investigating the impact of unemployment seem to focus on primary or hospital care, but a broader assessment of other care settings is needed. This would help policymakers inform health care planning by determining whether there is some degree of complementarity or substitution between care settings. For example, unemployment may potentially increase the number of general practitioner (GP) visits as well as the demand for mental health services [6].

Third, little is known about what factors drive the relationship between unemployment and health care utilisation. For example, it is unclear whether changes in health care utilisation are driven by wider individual and societal factors, other than through changes in health itself. Identifying these factors and pathways of impact can help policymakers tailor health policies to minimise the impacts of unemployment or job insecurity on health care utilisation [7]. This may involve, for instance, developing health prevention and promotion policies in the workplace. In addition, unemployment and job insecurity are likely to exacerbate inequalities in health, and understanding how these might interact with other known drivers of inequalities is important. For example, a recent study in Germany showed that unemployed individuals faced access barriers to the health care system [8].

This review seeks to address these gaps in knowledge by critically appraising and summarising the published literature on the impact of unemployment and job insecurity on health and social care utilisation. This study aims to clarify: (1) the direction and magnitude of the reported effects of unemployment and job insecurity on health and social care utilisation, (2) whether the impact differs according to care setting, and (3) the main factors driving the relationship between unemployment and service use.

## Methods

The protocol of this review was registered in PROSPERO (CRD42020177668).

### Search strategy

We searched studies published between January 2000 and April 2021. Databases of MEDLINE, Scopus, Web of Science, PsycINFO, Embase and CINAHL PLUS were searched; we combined search terms related to ‘unemployment/job insecurity’ and ‘health/social care utilisation’ to capture relevant studies (full search strategy shown in Appendix 1. For the purposes of this review, we broadly defined ‘health care utilisation’ as the quantification of individual use of health services to prevent, diagnose and treat health conditions, and maintain an individual’s health and well-being [9, 10]. This encompasses services across primary, secondary, mental health and social care settings [11]. Primary care is usually the first point of contact for patients, while secondary care includes hospital care and other specialist services referred by primary care providers [11]. Mental health care distinctively targets mental health conditions and mainly includes medication and specialist services. Social care supports broader (non-health) needs of individuals that stem from illness and disability [12].

### Inclusion and exclusion criteria

In general, we included studies that reported an association between unemployment and/or job insecurity in health and social care utilisation. We excluded studies that: (i) explored individual preferences (in which being unemployed might be one of the drivers) between different health services, (ii) relied on aggregate data, such as unemployment rates, (iii) were not published in English, and (iv) papers published before 2000, as a review published in the 1990s [13] suggested scant evidence on the impact of unemployment on healthcare utilisation until that point.

### Screening and data extraction

Two reviewers individually screened all titles and abstracts. Full-text screening was conducted by the first reviewer, with the second reviewer screening 20% of these. Any disagreements on inclusion were discussed and resolved with a third reviewer. For each individual study, we extracted key information on population size, exposure, comparator, outcomes and confounders. We used narrative synthesis to summarise the direction of the impact, and the factors driving the association between unemployment and health care utilisation.

### Meta-analysis

We conducted a meta-analysis to synthesise the impact of unemployment on health service use, for a subset of studies with similar exposures, comparators and outcome measurements. Studies were included in the meta-analysis if they: (i) compared service use between unemployed and employed individuals, and (ii) reported odds ratio as a measurement of association. We used a random-effects model that allows for the observed estimates of the impact of unemployment to vary across the included studies [14], due to observed differences between studies (rather than chance). Such heterogeneity (measured by the I-squared) among the studies can be a result of differences in the study design (e.g., study population and follow-up) or contextual differences, such as the health care system and labour market. Given that some studies reported more than one effect size, we repeated the meta-analysis using a robust variance estimation [15] to adjust for within-study correlation. The meta-analysis was conducted using the metan package in STATA.

### Risk of bias

We assessed the risk of bias in each study using the ROBINS-I checklist [16], which evaluates the risk of bias based on four domains: cofounding, selection, information and reporting bias (Appendix 2.1). The risk of bias was categorised into ‘low’ (L), ‘moderate’ (M), ‘serious’ (S), ‘critical’ (C), or no information (NI). Studies with two or more domains judged S or C were at serious risk of bias. The aim of this assessment was to gauge overall quality of the published studies, but no studies were excluded based on their risk of bias.

## Results

### Included studies

**Fig. 1:**
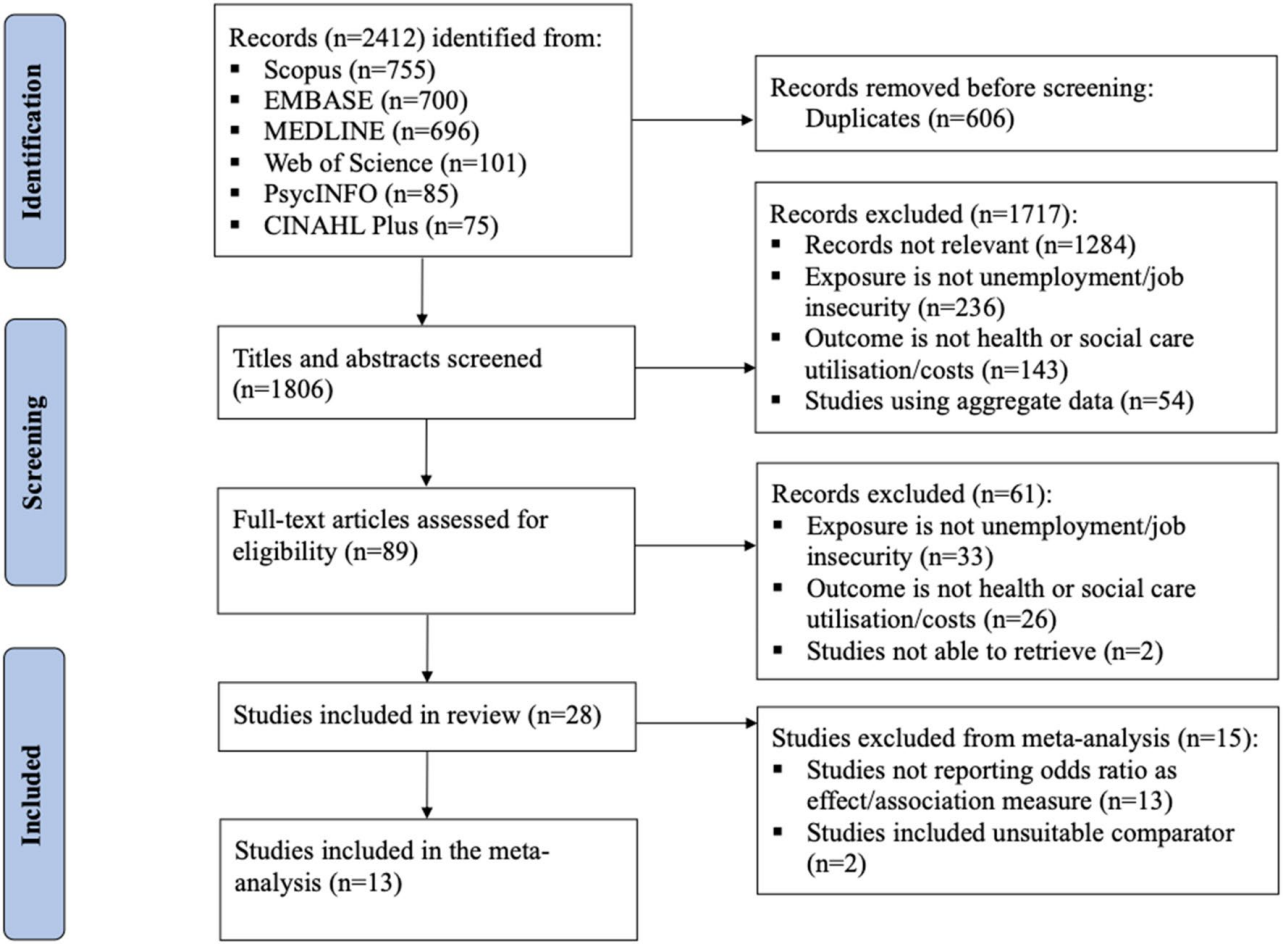
PRISMA flowchart

The review included 28 studies, and 13 of these (marked in Appendix 3) were eligible for the meta-analysis as illustrated in Fig. 1. Among the included studies, sample size varied significantly, ranging from 243 to 3,284,896 of participants (further details in Appendix 3). Studies were conducted across a wide range of countries, with a large proportion conducted in the European countries (*n* = 12) followed by the United States (*n* = 4) and Australia (*n* = 4). Also, the included studies covered different types of health care systems including systems funded through taxation, social, and private health insurance. Among the included studies, three different care settings were identified. First, the primary care setting (*n* = 15), which refers to patient’s first point of contact, comprises GP visits, preventive care such as maternal care and general health check-up. Hospital care (*n* = 11), the second care setting, includes hospitalisation and specialist visits. The last care setting identified is mental health care (*n* = 5) which includes mental health consultation and prescription. In addition, there were studies (*n* = 3) that reported mixed (among the three) care settings.

### Direction of the impact

Direction of the impact in this review refers to the way that unemployment is associated with health service use; for example, a positive impact means that the unemployed are associated with higher use of health services compared to the employed. Table 1 reports the total number of effect size estimates (*N*) and studies (n in parentheses) on the impact of unemployment and job insecurity on service use in the care setting. In total, 79 estimates were identified from the 28 included studies. Just over 50% (*N* = 41) of the effect sizes reported a positive association between unemployment/job insecurity and health care utilisation across all care settings, and the estimates were distributed to a wide range of studies (*n* = 22, 79% of the included studies).

**Table 1:** Direction of the impact by care settings

	Service type	Positive association *N* (*n*) ^¶^	Negative association *N* (*n*)	No association* N*(*n*)	Total
Primary care	GP visits	11 (9)	5 (2)	3 (2)	19 (10)
	Prescription (from GP)	1 (1)	0	2 (1)	3 (1)
	Women’s health	0	2 (2)	1 (1)	3 (3)
	Total health expenditure	13 (1)	5 (1)	0	18 (1)
	Total	25 (11)	12 (5)	6 (4)	43 (15)
Hospital care	Emergency	1 (1)	0	1 (1)	2 (2)
	Non-emergency	4 (4)	4 (2)	12 (4)	20 (10)
	Total	5 (5)	4 (2)	13 (5)	22 (11)
Mental health care	General mental health service	2 (1)	2 (2)	0	4 (2)
	Psychiatric visits	1 (1)	0	0	1 (1)
	Prescription	5 (2)	0	1 (1)	5 (3)
	Total	8 (4)	2 (2)	1 (1)	11 (5)
Mixed care ^¶¶^	Primary and hospital care	1 (1)	0	0	1 (1)
	Primary and social care	1 (1)	0	0	1 (1)
	Primary, social community and hospital care	1 (1)	0	0	1 (1)
	Total	3 (3)	0	0	3 (3)
Total		41 (22)	18 (7)	20 (10)	79 (28)

^¶^*N*—number of estimates; *n*—number of studies. Please note that some studies may report estimates from different settings and more than one type of association, and hence, the number of studies may not add up exactly to the total in the last row, or in the last column. Also, a positive association indicates that being unemployed/under job insecurity is associated with more health service use

^¶¶^Studies within the mixed care category only report one general estimate comprising more than one care setting

In primary care setting, there was considerable uncertainty even with more prevalent positive associations (*N* = 25 out of 43). Over half of the estimates (*N* = 11 out of 19) reported a positive association with GP visits, and 72% of estimates (*N* = 13 out of 18) resulted from the same association in total health expenditure. However, the former was distributed to nine studies (out of 10), while the later was only dominated by one study. Women’s health had two out of three estimates (and studies) reported a negative association with unemployment.

About 60% of estimates (*N* = 13 out of 22) found no association between unemployment and hospital care. In addition, majority of estimates (*N* = 20 out of 22) in hospital care are non-emergency care among which more than half of them (*N* = 12 out of 20) found no association with unemployment.

Positive association (*N* = 8 out of 11) dominates in mental health care settings; five positive estimates in prescription, two in general mental health service, and one in psychiatric visits, respectively. The only two estimates that found a negative association belong to general mental health care, while the only one estimate found no association between unemployment and prescription for mental health.

All estimates in mixed care settings reported a positive association, meaning that the unemployed or people under job insecurity tend to use more mixed care in general. Nevertheless, none of the studies examine unemployment effects on community or social care utilisation separately from the other care settings.

Only two included studies considered the impact of job insecurity on health service use; one reported a negative association with primary and hospital service use, and the other reported a positive association with mental health service use (further details in Appendix 3).

### Magnitude of the impact

**Fig. 2:**
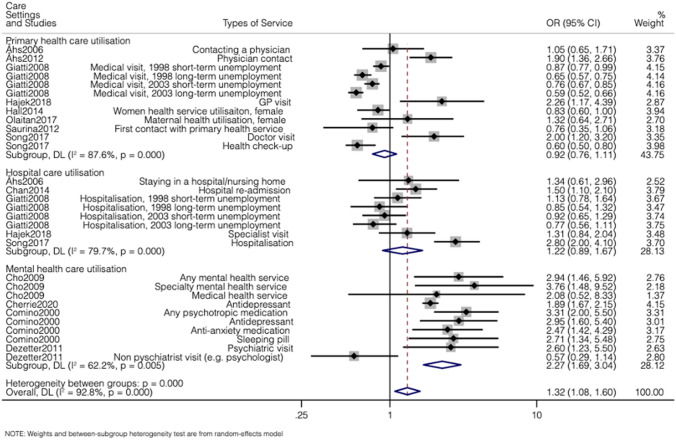
Association between unemployment and health care utilisation according to care setting

Among the studies included in the meta-analysis, the sample sizes vary from 243 to 32,887 and they were conducted across a wide range of countries. These studies considered the impact of unemployment across primary, hospital and mental health care utilisation. Four studies [4, 17–19] with 15 estimates reported on both primary and hospital service use; four studies [20–23] with four estimates reported only on primary service use, and one study [24] with one estimate considered hospital care utilisation. Four separate [6, 25–27] studies with 10 estimates reported unemployment impacts on mental health service. Figure 2 describes the results of the meta-analysis, reported in health care setting. Overall, the pooled odds ratio across all care settings is 1.32 (95% CI 1.08, 1.60), suggesting that unemployed individuals are on average 30% more likely to use health care services compared to employed individuals. This was largely driven by the impact of unemployment on mental health services, where the odds ratio was 2.27 (95% CI 1.69, 3.04). There was no association between unemployment and primary care utilisation (odds-ratio was 0.92, 95% CI 0.76, 1.11) or hospital care utilisation (odds ratio was 1.22, 95% CI 0.89, 1.67). In appendix 4, pooled effects were reported by different health financing systems. Studies conducted in countries with social health insurance reported the highest pooled odds ratio, 1.95 (95% CI 1.35, 2.83), but this was again largely dominated by mental health care utilisation.

The between-study heterogeneity, measured by the I-squared, is high across all care settings (above 60%). The overall findings of the meta-analysis remained similar after implementing the robust variance approach (Appendix 5), with the pooled odds ratio being 1.39 (95% CI 1.08, 1.80).

### Potential drivers of the impact

Only nine studies explored potential factors affecting the relationship between unemployment (or job insecurity) and health service use. In general, two different levels of factors were identified.

The first level includes system factors, where healthcare system plays a vital role. Five studies [5, 17, 18, 22, 28] suggested that health system with high out-of-pocket payment and lack of health insurance may have explained the negative impact of unemployment and job insecurity on primary and hospital care utilisation. However, even in health systems with universal coverage, people with private health plans (usually provided by employers) tend to have greater access to health care, leading to less health service use among precarious workers and the unemployed [18]. Moreover, people losing their jobs are likely to lose their private health plan provided by employers, thus cutting health spending even with needs. Therefore, the health system can affect the impact of unemployment and job insecurity on health service use in different ways.

The second level of factors is individual-specific drivers, such as individuals’ well-being, social network and disposable time. Four studies [5, 6, 17, 29] found that increased use in mental health services was mostly driven by depression/anxiety, as a result of unemployment. Åhs, Burell & Westerling [20] reported that unemployment was associated with a smaller social network, and that potentially led to fewer doctor visits through recommendations from friends or colleagues. Both Åhs, Burell & Westerling [20] and Mayer & Österle [30] found that more disposable time amongst unemployed individuals drove an increase in primary and hospital care utilisation.

### Risk of bias

The overall risk of bias was low, suggesting relatively high quality of the included studies. More than half of the included studies (*n* = 18) were judged to have low risk of bias (full details reported in Appendix 2.2). None of the studies are associated with a critical risk of bias. Only two studies [4, 5] (one included in the meta-analysis) were considered seriously biased, for example due to potential selection bias (sample selection and missing data issues), and information bias due to poorly defined exposure. Meta-analysis results remained similar when the seriously biased study [4] was excluded.

## Discussion

This review identified 28 studies with 79 estimates of the impact of unemployment and job insecurity on health service use. Most estimates reported a positive impact of unemployment and job insecurity on mental health service use, but the impact on primary and hospital service use is more ambiguous. The results from the meta-analysis suggested that unemployed individuals are approximately 30% more likely to use health service, which is largely explained by mental health service use. The reviewed studies suggested that both system and individual-specific factors may affect the relationship between unemployment and health care utilisation. The system-level factor mainly refers to the health system, and it may change the relationship between unemployment and health service use in different ways. For example, unemployed individuals might use more health service in countries with universal health coverage and less out-of-pocket payment, while job loss could potentially reduce health service use in countries where the health insurance is mostly provided by employers. On the other hand, individual-level factors including more disposable time, less social networking opportunities and higher risk of depression caused by unemployment or job insecurity tend to increase the use of health services. Based on the results of ROBINS-I checklist assessment, the majority of the included studies were associated with a low risk of bias.

This is the first comprehensive review of the impact of unemployment on health care utilisation, and it makes several contributions to the literature. First, this paper finds a positive impact of unemployment and job insecurity on health service use; it reviews the impact of unemployment differs across health care settings and finds that the impact of unemployment is more pronounced on mental health service use, but it is likely to affect primary care services as well. Second, it finds that the impact on care utilisation is likely to be driven not only by changes in health needs, such as deteriorating mental health, but also changes in financial circumstances, health insurance and disposable time. The finding of different drivers partly unveils the complexity of the relationship between unemployment and health service use; not only health system but also individual differences such as disposable time and networking opportunities affect their relationship. Third, in addition to unemployment, two studies [23, 29] identified in this paper found health service use is associated with job insecurity, which has been arguable of greater concern to policymakers in the last few years. Fourthly, this review considers the impact of unemployment on both health and social care utilisation and finds the latter has received little attention so far.

The key findings from our review are in line with those from the previous review [13]. That review focused mostly on the impact of unemployment on health, but it has also reported some evidence of a positive association between unemployment and health care utilisation, including mental health services. However, most studies included in that review were based on aggregate data (e.g., impact of unemployment rates on hospital admissions). By focusing on individual-level data, combined with the relatively low risk of bias of the studies included, our review provides more in-depth evidence of the impact of unemployment across different health care settings. The previous review has also hinted that this positive impact of unemployment might not be transferrable to health financing systems based on private insurance, such as the United States, because hard economic times may mean less ability to pay for health care. Our meta-analysis included only one study conducted in the United States, which suggests that unemployed women are less likely to seek maternal health services. Appendix 4 reports the meta-analysis results according to the type of healthcare financing system.

Our review sheds some light on potential pathways of impact of unemployment on health care utilisation. Some studies [5, 6, 17, 18, 20, 22, 28–30] explored the extent to which economic factors, education and social supports affected the relationship between unemployment and health service use. For example, other things being equal, unemployed individuals in lower-income groups were relatively less likely to seek health care services compared to higher income unemployed individuals. To better meet the needs of the unemployed, policymakers will need to pay particular attention to the more deprived unemployed population, because these may themselves be less likely to seek care despite the perceived need, and may face barriers (e.g., user fees, digital exclusion) to access health care. Another important consideration for policymakers is the need to improve the preparedness of the health care system to handle mental health needs of unemployed in a way that does not affect the prospects of future employment. For example, for the same symptoms (e.g., anxiety), unemployed individuals are more likely to be prescribed antidepressants (often leading to addiction and abuse) compared to the employed [6].

This review has some limitations. First, a few studies explored potential factors driving the relationship between unemployment and health care utilisation, but none of the studies was able to make causal claims about either the overall effects of unemployment or potential ‘mediators’ of the effect. Thus, this review is unable to uncover a cause–effect relationship between unemployment and healthcare utilisation and/or establish potential mediators of this impact. Second, the meta-analysis included less than half of the reviewed studies, which affected particularly studies in primary care, because they often reported correlation coefficients (instead of odds ratios). Coincidentally, proportionately fewer of these studies in primary care reporting a positive impact were included (3 out of 11) in the meta-analysis, compared to those reporting a negative effect (3 out of 5). This meta-analysis has underestimated the positive impact of unemployment on primary care utilisation reported in Fig. 2. Third, it is possible that some differences across studies may have been explained by broader country-specific differences, for example cultural and lifestyle aspects, but the review is not able to fully disentangle this. Four, this review has not included grey literature, so may have missed some unpublished reports on the topic, although the methodological quality of these is likely to be poorer than that of peer-reviewed studies.

This review has identified several areas for further research. First, most of the reviewed studies included only one care setting. However, considering a wider range of settings will enable us to have a better understanding of service use implications of unemployment across different settings. For instance, social care utilisation is rarely mentioned by the included studies. However, unemployed individuals often seek help from social workers [31, 32], and hence, the necessity to investigate effects of unemployment in this setting is warranted. Second, the analysis of different drivers combined with Andersen’s behaviour model of health care utilisation could be further explored. None of the included studies explored how the wider system and individual-level factors, such as macroeconomic situation and an individual’s ethnicity, interact and affect the impact of unemployment on health service use [33, 34]. In addition, more methodologically rigorous studies for exploring potential non-health factors between unemployment and health care utilisation are needed [35]. Third, while the methodological quality of the included studies is reasonably good, most of the papers focused on examining the associational, rather than a causal effect of unemployment on health care utilisation. Therefore, those papers are limited to examine the possibility of reverse causality, where people with more health needs are less likely to be employed. This is mainly due to the data limitations, but recent studies suggested that better evidence combined with improved research designs may help enable the estimation of the causal effects of unemployment [36–38].

In summary, a working day appears to keep the doctor of mental health away, but does not necessarily reduce primary or hospital care visits. Future work to examine the impact of unemployment across other care settings, including community and social care, and to further explore non-health determinants of utilisation should be prioritised.

## Supplementary Information

Below is the link to the electronic supplementary material.

Supplementary file1 (DOCX 1137 KB)
