# Supplementary material for: Does a working day keep the doctor away? A critical review of the impact of unemployment and job insecurity on health and social care utilisation
Source: Eur J Health Econ. 2022 May 6;24(2):179–86. doi: 10.1007/s10198-022-01468-4 (PMC9985560; doi:10.1007/s10198-022-01468-4)
Supplement: Supplementary file 1 — Supplementary file1 (DOCX 1137 KB) [file 10198_2022_1468_MOESM1_ESM.docx]

# Appendices

## Appendix 1. Searching strategy on Medline

1. unemployment/

2. (unemploy* or "job loss" or "loss of job" or "lost job" or "work loss" or "loss of work" or "lost work" or "jobless" or "job seek*" or "out of work" or "out of employment" or "not employed").mp. [mp=title, abstract, original title, name of substance word, subject heading word, floating sub-heading word, keyword heading word, organism supplementary concept word, protocol supplementary concept word, rare disease supplementary concept word, unique identifier, synonyms]

3. 1 or 2

4. ("employment status" or "job *secur*" or "job *stab*" or "temporary job" or "employment *security" or "*secure employment" or "*security of employment" or "employment *stab*" or "*stable employment" or "*stability of employment" or "temporary employment" or "short-term work" or "temporary work" or "short-term employment").mp. [mp=title, abstract, original title, name of substance word, subject heading word, floating sub-heading word, keyword heading word, organism supplementary concept word, protocol supplementary concept word, rare disease supplementary concept word, unique identifier, synonyms]

5. 3 or 4

6. exp Health Services/

7. ("health care use" or "use of health care" or "health care utili?ation" or "utili?ation of health care" or "health care consumption" or "health service use" or "use of health service" or "health service utili?ation" or "utili?ation of health service" or " health resource utili?ation" or "utili?ation of health resource" or "social care use" or "use of social care" or "social care utili?ation" or "utili?ation of social care" or "social service use" or "use of social service" or "social service utili?ation" or "utili?ation of social service" or "use of long-term care" or "use of day care").mp. [mp=title, abstract, original title, name of substance word, subject heading word, floating sub-heading word, keyword heading word, organism supplementary concept word, protocol supplementary concept word, rare disease supplementary concept word, unique identifier, synonyms]

8. 6 or 7

9. ("health care cost" or "cost of health care" or "health care expenditure" or "expenditure on health care" or "health service cost" or "cost of health service" or "health service expenditure" or "expenditure on health service" or "cost of medicine" or "medical cost" or "medical expenditure" or "primary care cost" or "cost of primary care" or "cost of mental health" or "dental care cost" or "cost of dental care" or "social care cost" or "cost of social care" or "social care expenditure" or "expenditure on social care" or "social service expenditure" or "expenditure on social service").mp. [mp=title, abstract, original title, name of substance word, subject heading word, floating sub-heading word, keyword heading word, organism supplementary concept word, protocol supplementary concept word, rare disease supplementary concept word, unique identifier, synonyms]

10. ("health care visit" or "consultation" or "medication" or "doctor visit" or "GP visit" or "psychiatric visit" or "outpatient visit" or "inpatient visit" or "hospital visit" or "hospital admission" or "specialist visit" or "specialist referral" or "antidepressant use" or "use of antidepressant" or "help-seeking" or "social service visit").mp. [mp=title, abstract, original title, name of substance word, subject heading word, floating sub-heading word, keyword heading word, organism supplementary concept word, protocol supplementary concept word, rare disease supplementary concept word, unique identifier, synonyms]

11. 9 or 10

12. 5 and 8 and 11

## Appendix 2

### Appendix 2.1. Risk of bias assessment criteria

| ***Bias Domain*** | ***C*** | ***S*** | ***M*** | ***L*** | ***NI*** |
| --- | --- | --- | --- | --- | --- |
| *1.Confounding bias* | | | | | |
| ***Bias due to confounding*** | No controls for confounding factors | Limited confounding factors are controlled (e.g. only sex and age) | A wide range of confounders are controlled but within socioeconomic factors; limited confounding factors are controlled but beyond socioeconomic factors | Controlled beyond socioeconomic factors | No information |
| *2.Selection bias* | | | | | |
| ***Bias in selection of participants into the study and missing data*** | Sample not from general population without adjustments; poor response rate; some outcome data are missing; part of participants’ data is missing (incomplete survey) and no methods applied to account for missing data | Sample not from general population with appropriate adjustments; high/moderate response rate; some outcome data are missing; part of participants’ data is missing (incomplete survey) and methods applied to account for missing data | Sample from general population with exclusion of some eligible participants; unsatisfactory response rate; outcome data are mostly complete; part of participants’ data is missing (incomplete survey) and methods applied to account for missing data | Sample from general population and high response rate if survey conducted; outcome data are mostly complete; only small portion of participants’ data missing for same reason across groups and appropriate methods applied to account for missing data | No information |
| *3.Information bias* | | | | | |
| ***Bias in measurement of interventions and outcome*** | Self-reported data without a definition/guideline OR no definition of the exposure; vague classification of the exposure; self-report outcome only without any procedures and instructions illustrated | Poorly defined and classified exposure (e.g. no clarification on period of unemployment included and the classification is simply the employed/unemployed); Self-report outcome only with procedures and instructions illustrated | Self-reported exposure status with a clear definition/guideline; a clear classification of the exposed group; Self-report outcome validated by administrative data; followed by a valid and justified procedures and instructions | Clearly define and classify participants’ exposure status (unemployment or job insecurity); outcome measured by valid and consistent procedures and instructions or using administrative data | No information |
| *4. reporting bias* | | | | | |
| ***Bias in selection of the reported result (reporting bias)*** | Reported outcomes are completely inconsistent with prespecified outcomes; most subgroups are missing | Only some of the prespecified outcomes are reported; certain subgroups are missing in the outcome | Most pre-specified outcomes are reported; most subgroups are well discussed | All pre-specified outcomes are reported; all subgroups are well discussed | No information |
| *Note: C = critical risk of bias; S = serious risk of bias; M = moderate risk of bias; L = low risk of bias; NI = no information | | | | | |

### Appendix 2.2 Risk of bias results

| Study | Confounding bias | Selection bias | Information bias | Reporting bias | Overall bias |
| --- | --- | --- | --- | --- | --- |
| Åhs2006 | L | M | L | S | M |
| Åhs2012 | M | S | M | M | M |
| Arruda2016 | M | M | M | L | L |
| Bonizzato2000 | NI | M | M | L | L |
| Chan2014 | NI | M | S | M | M |
| Cherrie2020 | N | N | N | N | L |
| Chiavarini2014 | M | L | L | L | L |
| Cho2009 | M | M | M | L | L |
| Collie2021 | N | M | N | N | L |
| Comino 2000 | L | L | M | L | L |
| Cumming2010 | L | L | L | L | L |
| Dezetter2011 | M | M | M | L | L |
| Elser2019 | M | L | L | L | L |
| Geyer2003 | S | S | M | L | S |
| Giatti2008 | L | L | M | L | L |
| Hajek2018 | NI | S | S | L | S |
| Hall2014 | M | M | M | L | L |
| Hollander2013 | M | L | L | L | L |
| Martindale2016 | NI | S | M | M | M |
| Mayer2015 | L | M | M | M | L |
| Moustgaard2014 | L | S | M | L | M |
| Nolan2007 | M | M | M | L | L |
| Olaitan2017 | NI | S | M | L | M |
| Saurina2012 | M | M | S | L | M |
| Schaller2015 | L | M | L | L | L |
| Schofield2012 | NI | L | L | M | L |
| Song2017 | S | M | M | L | M |
| Vedsted2005 | L | M | M | L | L |

 *Note: S = serious risk of bias; M = moderate risk of bias; L = low risk of bias; NI = no information

## Appendix 3. Key characteristics of the reviewed studies.

| **Study** | **Country** | **Sample size** | **Exposure** | **Comparator** | **Resource use/cost**  **category** | **Odds ratio (95% CI)** | **Adjustment** |
| --- | --- | --- | --- | --- | --- | --- | --- |
| * Åhs & Westerling , 2006 [1] | Sweden | 2,648 | Unemployment | The employed | Hospital service use:  1) contacting a physician  2) staying in a hospital/nursing home | 1) 1.05 (0.65 – 1.71)  2) 1.34 (0.61 – 2.96) | Age, gender, country of birth, educational level and cohabiting status, economic situation, social network and support, long-standing illness or handicap |
| * Åhs, Burell and Westerling, 2012 [2] | Sweden | 1,211 | Unemployment | The employed | Primary service use: physician contacts | 1.90 (1.36–2.66) | Sex, age, country of birth, educational level, marital status, social network, economic hardship |
| Arruda & Marcon, 2016 [3] | Brazil | 421 | Unemployment | Employer/self-employed/  employee | Primary and hospital service use | 3.06 (1.06 - 8.86) | Age range, skin colour, marital situation and education |
| Bonizzato et al., 2000 [4] | Italy | 543 | Unemployment | The employed | Primary, hospital and, social and community service costs | *Linear regression coefficient:  0.217 (0.023 - 0.412) | Weighting adjustments made for compensating for non-response |
| * Chan & Wong, 201) [5] | Singapore | 1,509 | Unemployment | The retired, housewife and others | hospital readmission | 1.5 (1.1 - 2.1) | Predisposing, enabling, needs and health factors |
| * Cherrie et al., 2020  [6] | The United Kingdom | 8,025 | Unemployment | The employed | Mental health service use: antidepressant prescriptions | Inverse of no prescriptions: 1.887 (1.667 - 2.174) | Age, sex, Carstairs deprivation, employment status, ethnicity, social grade, living alone, and marital status |
| Chiavarini et al., 2014 [7] | Italy | 37,000 | Unemployment | Self-employed or white-collar workers | Primary service use: prenatal care | Low level of prenatal care use:  1.56 (1.29-1.88) | Regional difference |
| * Cho et al., 2009 [8] | Korea | 243 | Unemployment | The employed | Mental health services use:  Any mental health services  Specialty health services  Medical services | 1) 2.94 (1.46 - 5.92)  2) 3.76 (1.48 - 9.52)  3) 2.08 (0.52 - 8.33) | Age and gender |
| Collie, Sheehan & McAllister , 2021 [9] | Australia | 9,110 | Unemployment | Wage earners | Primary service use:  1) General practitioner  Hospital service use:  2) Specialist visits  3) Admitted to hospital  4) Visited Emergency  5) Dentist visit  6) Medication  7) Supplements | Incidence rate ratios  1) 1.11 (1.02 – 1.20)  2) 1.09 (0.87 – 0.87)  3) 1.21 (0.89 – 1.66)  4) 0.95 (0.68 – 1.30)  5) 1.03 (0.87 – 1.22)  6) 1.12 (0.99 – 1.27)  7) 0.93 (0.79 – 1.11) | Age, sex, insured status, financial reserves, self-assessed health and disability status |
| * Comino et al., 2000 [10] | Australia | 2,665 | Unemployment | The employed | Primary service use:  1) Any psychotropic medication  2) Antidepressant  3) Anti-anxiety  4) Sleeping pills | 1) 3.31 (2.0 - 5.5)  2) 2.95 (1.60 - 5.40)  3) 2.47 (1.42 - 4.29)  4) 2.71 (1.34 - 5.48) | Age, sex, non-English speaking background and severity. |
| Cumming et al., 2010 [11] | New Zealand | 19,506 | Unemployment | The employed | Primary service use:  1) Probability of GP visits  2) Number of GP visits  Hospital service use:  3) Possibility of secondary practitioner visits | * Marginal effect of  Fulltime employed  1) -0.04  2) -0.705  3) -0.069 | Health insurance status, self-reported health status, chronic health conditions and health behaviours |
|  |  |  |  |  |  |  |  |
| * Dezetter et al., 2011 [12] | Mixed European countries | 8,796 | Unemployment | Workers | Mental health service use | 1) Psychiatrist only vs. nonpsychiatrist only = 2.6 (1.23 - 5.50)  2) Non psychiatrist only vs. psychiatrist only = 0.57 (0.29 - 1.14) | Age and Gender |
| Elser et al., 2019 [13] | The United States | 15,502 | Job insecurity | The laid off | Mental health service use  Outpatient visits  Prescriptions | * Multivariate adjusted beta:  1) 0.01 (0.004-0.017)  2) 0.012(-0.002 - 0.026) | Sex, age, race/ethnicity (Black, Hispanic, white, other), terciles of hire year |
| Geyer & Peter, 2003 [14] | Germany | 105,554 | Unemployment | The employed | Hospital service use: hospital admission | *  Relative risk ratio  Unemployed 2-8 months= 0.31(0.28 - 0.34)  Unemployed 7-16 months= 0.35(0.32 - 0.39)  Unemployed 17-24 months= 0.28(0.23 - 0.33) | Occupation |
| * Giatti Barreto and César, 2008 [15] | Brazil | 31,870 in 1998 and 32,887 in 2003 | Unemployment | Full time employment with social protection | Primary service use:  Medical visits  Hospitalisation in past 12 months | 1998/2003  1) Short term = 0.87 (0.77 – 0.99) / 0.76 (0.67 – 0.85)  Long term = 0.65 (0.57 – 0.75) / 0.59 (0.52 – 0.66)  2) Short-term = 0.87 (0.77 – 0.99) / 0.92 (0.65 – 1.29)  Long term = 0.85(0.54 – 1.32) / 0.77 (0.56 – 1.11) | Age, schooling and chronic disease |
| * Hajek & König, 2018 [16] | Germany | 1,049 | Unemployment | The employed | Primary health services:  GP visits  Hospital care use:  Specialist visits | 1) 2.26 (1.17 - 4.39)  2) 1.31 (0.84 - 2.04) | No information |
| * Hall et al., 2014 [17] | The United States | 7,897 | Unemployment | The employed | Primary service use | 1.2 (1.0 - 1.5) (the employed) | No information |
| Hollander et al., 2013 [18] | Sweden | 3,284,896 | Unemployment | The employed | Hospitalisation | *Unadjusted hazard ratio  1.94 (1.85-2.04) | Age group, marital status and education |
| Martindale-Adams et al., 2016 [19] | The United States | 642 | Unemployment | The employed | Primary and social service use | *Linear regression coefficient  - 0.098 (being employed) | No information |
| Mayer & Österle, 2015 [20] | Australia | 13,291 | Unemployment | The employed | Primary service use:  Prescribed medicine  Minor polypharmacy  Major polypharmacy | *Relative risk rate  1) 1.34 (0.99-1.83)  2) 1.14 (0.76-1.71)  3) 1.98 (1.20-3.28) | Health indicators (self-assessed health, chronic conditions), demographic characteristics (age, sex) and outpatient visits |
| Moustgaard, Joutsenniemi and Martikainen, 2014 [21] | Finland | 231,629 | Unemployment | The employed | Hospital service use: psychiatric admissions | *Hazard ratio  1.19 (0.77 - 1.85) | Age, gender, region of residence, baseline depression severity, and psychiatric comorbidity |
| Nolan, 2007 [22] | Ireland | 49,237 | Job loss | The employed | Primary service use: GP visits | *Marginal effect  0.18 (0.06) | No information |
| * Olaitan et al., 2017 [23] | Nigeria | 371 | Unemployment | The employed | Primary service use: maternal care utilisation | 1.32 (0.64-2.71) | No information |
| * Saurina et al., 2012 [24] | Spain | 575 | 1) Unemployment  2)  Permanent contract | 1) Paid work  2) Temporary contract | Primary service use: frequency of visits | 1) 1.1 (1.006, 1.208)  2)  Permanent contract = 1.085 (0.991, 1.193)  Temporary contract = 1.075 (0.979, 1.185) | No information |
| Schaller & Stevens, 2015 [25] | The United States | 108,516 | Job loss | workers who never be displaced  a. before job loss  b. during job loss  c. after job loss | Primary service use and costs:  Office visits  Office visit costs  Emergency room visits  Emergency visit costs  Prescription use  Prescription costs | *Linear regression coefficient  1) a = 0.0253, b = 0.0282, c = 0.0120  2) a = -39.3156, b = 17.3551, c = 5.5475  3) a = 0.0051, b = 0.0056, c = 5.5475  4) a = 2.1800, b = 1.9820, c = -1.0085  5) a = -0.0203, b = 0.0183, c = 0.0097  6) a = -54.4244, b = -8.1899, c = -7.8546 | Baseline self-reported health and mental health and dummies for gender, race, and educational attainment categories in addition to age and calendar year ﬁxed effects–an approach that has been common in the previous literature. |
| Schofield et al., 2012 [26] | Australia | No information | Unemployment | Not in the labour force | Primary service use  1) 1 visit to GP  2) 2 visits to GP  3) 3 or more visits to GP | *Relative risk ratio  1) Employed = 0.78 (0.68 - 0.89) Unemployed = 0.99 (0.74 - 1.32)  2) Employed = 0.78 (0.58 - 1.05) Unemployed = 1.17(0.63 - 2.18)  3) Employed = 0.96 (0.57 - 1.63) Unemployed = 0.63 (0.19 - 2.12) | No information |
|  |  |  |  |  |  |  |  |
| * Song et al., 2017 [27] | China | 2,906 | Unemployment | The employed | Primary service use:  Doctor visits  Annual health check-ups  Hospital service use:  Hospitalisation | 1) 2.0 (1.2 - 3.2)  2) 2.8 (2.0 - 4.1)  3) 0.6 (0.5-0.8) | Age and gender |
| Vedsted and Olesen, 2005 [28] | Denmark | 2,211 | Unemployment | The employed | Primary service use: frequency of GP visits | *Adjusted prevalence rates  Male = 1.26 (1.13-1.40)  Female = 1.14 (1.02-1.29) | Age, sex, physical and psychological health variables |

Note: Studies with * are included in the meta-analysis

### Appendix 4. Meta-analysis results according to health care system.

###
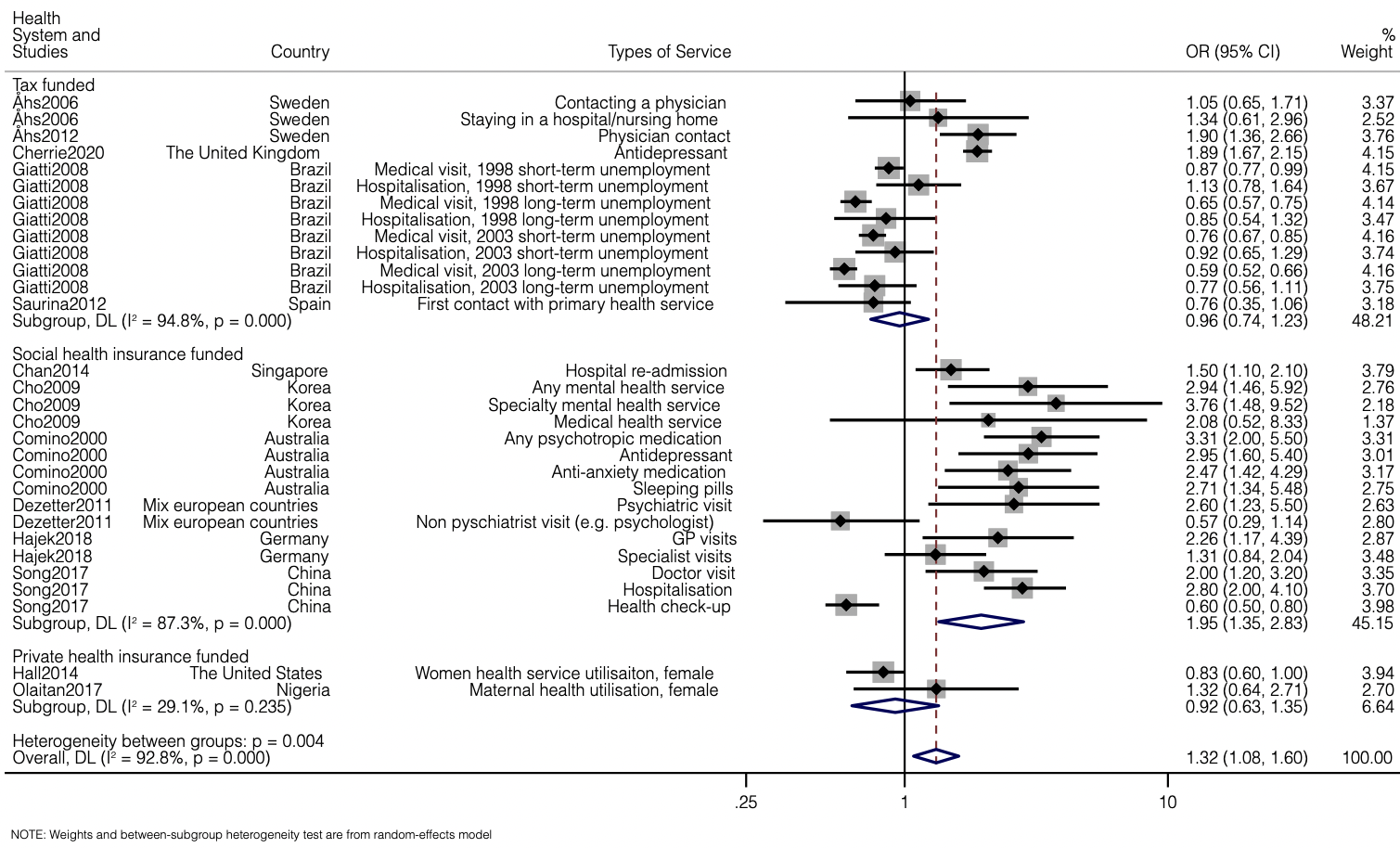


### Appendix 5. Meta-analysis results using a robust variance approach.

The robust variance approach reports on the log-odds ratio scale, therefore, the scale in forest plot below differs from that in Figure 2. The pooled log-odds ratio is 0.33 (95% CI: 0.075, 0588), and the corresponding odds ratio is 1.39 (95% CI: 1.08, 1.80).


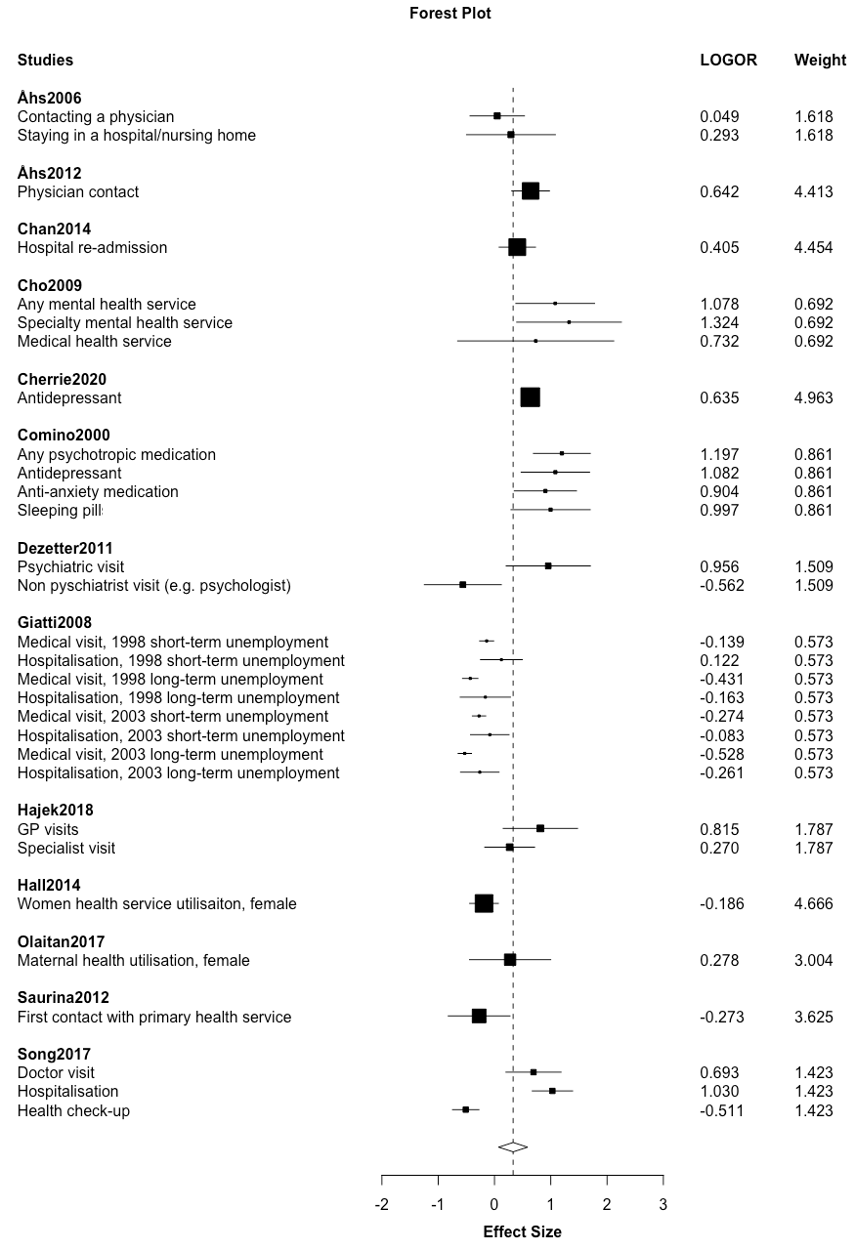


# Reference:

1. Ahs A.M., Westerling R.: Health care utilization among persons who are unemployed or outside the labour force. Health Policy. 78(2-3), 178-193 (2006)

2. Ahs A., Burell G., Westerling R.: Care or not care--that is the question: predictors of healthcare utilisation in relation to employment status. International Journal of Behavioral Medicine. 19(1), 29-38 (2012)

3. Arruda G.O., Marcon S.S.: Survey on the use of health services by adult men: prevalence rates and associated factors. Revista Latino-Americana de Enfermagem. 24, (2016)

4. Bonizzato P., Bisoffi G., Amaddeo F., Chisholm D., et al.: Community-based mental health care: to what extent are service costs associated with clinical, social and service history variables? Psychological Medicine. 30(5), 1205-1215 (2000)

5. Chan M.F., Wong F.K.: The risk factors for hospital re-admission in medical patients in Singapore. Health Soc Care Community. 22(5), 488-496 (2014)

6. Cherrie M., Curtis S., Baranyi G., McTaggart S., et al.: Use of sequence analysis for classifying individual antidepressant trajectories to monitor population mental health. BMC Psychiatry. 20(1), 551 (2020)

7. Chiavarini M., Lanari D., Minelli L., Salmasi L.: Socio-demographic determinants and access to

prenatal care in Italy. BMC Health Services Research. 24, (2016)

8. Cho S.J., Lee J.Y., Hong J.P., Lee H.B., et al.: Mental health service use in a nationwide sample of Korean adults. Social Psychiatry and Psychiatric Epidemiology. 44(11), 943-951 (2009)

9. Collie A., Sheehan L., McAllister A.: Health service use of Australian unemployment and disability benefit recipients: a national, cross-sectional study. BMC Health Services Research. 21(1), 249 (2021)

10. Comino E.J., Harris E., Silove D., Manicavasagar V., et al.: Prevalence, Detection and Management of Anxiety and Depressive Symptoms in Unemployed Patients Attending General Practitioners. Australian & New Zealand Journal of Psychiatry. 34(1), 107-113 (2000)

11. Cumming J., Stillman S., Liang Y., Poland M., et al.: The determinants of GP visits in New Zealand. Australian and New Zealand Journal of Public Health. 34(5), 451-457 (2010)

12. Dezetter A., Briffault X., Alonso J., Angermeyer M.C., et al.: Factors Associated With Use of Psychiatrists and Nonpsychiatrist Providers by ESEMeD Respondents in Six European Countries. Psychiatric Services. 62, 143-151 (2011)

13. Elser H., Ben-Michael E., Rehkopf D., Modrek S., et al.: Layoffs and the mental health and safety of remaining workers: a difference-in-differences analysis of the US aluminium industry. Journal of Epidemiology and Community Health (JECH). 73(12), 1094-1100 (2019)

14. Geyer S., Peter R.: Hospital admissions after transition into unemployment. Sozial und Praventivmedizin. 48(2), 105-114 (2003)

15. Giatti L., Barreto S.M., César C.C.: Informal work, unemployment and health in Brazilian metropolitan areas, 1998 and 2003. Cadernos de Saude Publica. 24(10), 2396-2406 (2008)

16. Hajek A., Konig H.H.: Which factors lead to frequent attendance in the outpatient sector among individuals in the second half of life? Evidence from a population-based longitudinal study in Germany. BMC Health Services Research. 18(1), 673 (2018)

17. Hall K.S., Dalton V., Johnson T.R.: Social disparities in women's health service use in the United States: a population-based analysis. Annals of Epidemiology. 24(2), 135-143 (2014)

18. Hollander A.C., Bruce D., Ekberg J., Burström B., et al.: Hospitalisation for depressive disorder following unemployment—differentials by gender and immigrant status: a population-based cohort study in Sweden. Journal of Epidemiology and Community Health. 67(10), 875-881 (2013)

19. Martindale-Adams J., Nichols L.O., Zuber J., Burns R., et al.: Dementia Caregivers' Use of Services for Themselves. Gerontologist. 56(6), 1053-1061 (2016)

20. Mayer S., Osterle A.: Socioeconomic determinants of prescribed and non-prescribed medicine consumption in Austria. European Journal of Public Health. 25(4), 597-603 (2015)

21. Moustgaard H., Joutsenniemi K., Martikainen P.: Does hospital admission risk for depression vary across social groups? A population-based register study of 231,629 middle-aged Finns. Social Psychiatry and Psychiatric Epidemiology. 49(1), 15-25 (2014)

22. Nolan A.: A dynamic analysis of GP visiting in Ireland: 1995-2001. Health Econ. 16(2), 129-143 (2007)

23. Olaitan T., Okafor I.P., Onajole A.T., Abosede O.A.: Ending preventable maternal and child deaths in western Nigeria: Do women utilize the life lines? PLoS One. 12(5), (2017)

24. Saurina C., Vall-Llosera L., Saez M.: Factors determining access to and use of primary health care services in the Girona Health Region (Spain). The European Journal of Health Economics 13(4), 419-427 (2012)

25. Schaller J., Stevens A.H.: Short-run effects of job loss on health conditions, health insurance, and health care utilization. Journal of Health Economics. 43, 190-203 (2015)

26. Schofield D.J., Shrestha R.N., Callander E.J.: Access to general practitioner services amongst underserved Australians: a microsimulation study. Human Resources for Health. 10(1), (2012)

27. Song X., Zou G., Chen W., Han S., et al.: Health service utilisation of rural-to-urban migrants in Guangzhou, China: does employment status matter? Tropical Medicine & International Health. 22(1), 82-91 (2017)

28. Vedsted P., Olesen F.: Social environment and frequent attendance in Danish general practice. British Journal of General Practice. 55, 510–515 (2005)
